# Drift in Neural Population Activity Causes Working Memory to Deteriorate Over Time

**DOI:** 10.1523/JNEUROSCI.3440-17.2018

**Published:** 2018-05-23

**Authors:** Sebastian Schneegans, Paul M. Bays

**Affiliations:** University of Cambridge, Department of Psychology, Cambridge CB2 3EB, United Kingdom

**Keywords:** computational modeling, population coding, saccadic eye movements, visual working memory

## Abstract

Short-term memories are thought to be maintained in the form of sustained spiking activity in neural populations. Decreases in recall precision observed with increasing number of memorized items can be accounted for by a limit on total spiking activity, resulting in fewer spikes contributing to the representation of each individual item. Longer retention intervals likewise reduce recall precision, but it is unknown what changes in population activity produce this effect. One possibility is that spiking activity becomes attenuated over time, such that the same mechanism accounts for both effects of set size and retention duration. Alternatively, reduced performance may be caused by drift in the encoded value over time, without a decrease in overall spiking activity. Human participants of either sex performed a variable-delay cued recall task with a saccadic response, providing a precise measure of recall latency. Based on a spike integration model of decision making, if the effects of set size and retention duration are both caused by decreased spiking activity, we would predict a fixed relationship between recall precision and response latency across conditions. In contrast, the drift hypothesis predicts no systematic changes in latency with increasing delays. Our results show both an increase in latency with set size, and a decrease in response precision with longer delays within each set size, but no systematic increase in latency for increasing delay durations. These results were quantitatively reproduced by a model based on a limited neural resource in which working memories drift rather than decay with time.

**SIGNIFICANCE STATEMENT** Rapid deterioration over seconds is a defining feature of short-term memory, but what mechanism drives this degradation of internal representations? Here, we extend a successful population coding model of working memory by introducing possible mechanisms of delay effects. We show that a decay in neural signal over time predicts that the time required for memory retrieval will increase with delay, whereas a random drift in the stored value predicts no effect of delay on retrieval time. Testing these predictions in a multi-item memory task with an eye movement response, we identified drift as a key mechanism of memory decline. These results provide evidence for a dynamic spiking basis for working memory, in contrast to recent proposals of activity-silent storage.

## Introduction

Much recent research has been directed at explaining the effects of set size (number of memoranda) on recall errors in visual working memory (Bays and Husain, 2008; Zhang and Luck, 2008; Bays et al., 2009; Ma et al., 2014; van den Berg et al., 2014). Population coding models have been shown to accurately reproduce the changing patterns of error observed at different set sizes in reproduction tasks (Bays, 2014; Schneegans and Bays, 2017a), and unlike other approaches they offer a concrete neural mechanism to account for set size effects. They propose that total population activity is fixed (normalized), such that fewer spikes contribute to the encoding of each item as set size increases. This leads to greater variability in the recovered value when a single memorized feature is decoded from the population activity.

Less attention has been devoted to the effects of time on working memory fidelity. Increasing the retention interval has been observed to adversely affect recall performance (Phillips and Baddeley, 1971; Zhang and Luck, 2009; Barrouillet et al., 2012; McKeown and Mercer, 2012; Pertzov et al., 2013, 2017) (but see Magnussen and Greenlee, 1999). However, the exact nature of this effect and the underlying neural mechanisms are poorly understood.

**Figure 1.**
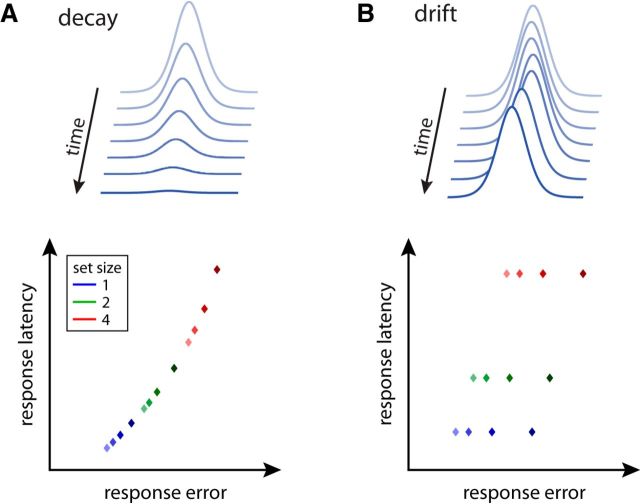
Delay effect models and behavioral predictions. ***A***, A feature value encoded in a population code can be visualized as a “hill” of activity centered on the stored value: the decay model assumes that this neural activity decreases over time (top). The expected effects of set size (indicated by color) and delay duration (indicated by different shades, with darker meaning longer duration) on response errors and latencies are shown (bottom). The decay model predicts that longer delays should produce an increase in both response error and latency, and that there should be a fixed relationship between these measures when both set size and delay duration are varied. ***B***, The drift model assumes that the activity distribution in the neural population undergoes random drift without change in total activity (top). It predicts that delay duration affects response errors, but not response latencies (bottom).

In the present work, we considered which changes in neural population activity could account for delay effects, and tested predictions derived from the population coding model in experiments. We contrasted two possible causes of decreasing recall performance with increasing delay duration. In a decay account, spiking activity is assumed to decrease continuously over the retention interval (Fig. 1*A*), analogous to the idea of memory traces decaying over time (Baddeley, 1986; Barrouillet and Camos, 2001; Barrouillet et al., 2007). Effects of delay in the neural population would then be directly comparable to the effects of set size, increasing recall variability by reducing the number of spikes contributing to the decoding of memorized features.

An alternative mechanism of delay effects is based on drift in the stored feature values (Kinchla and Smyzer, 1967; Nilsson and Nelson, 1981). In particular, attractor models of working memory (Camperi and Wang, 1998; Compte, 2006) describe how recurrent synaptic connections can create regions of sustained activity in neural populations. Due to noise in neural activity, these active regions will drift over time (Compte et al., 2000; Burak and Fiete, 2012; Wimmer et al., 2014), leading to gradual stochastic changes in the represented feature value (Fig. 1*B*). Importantly, total population activity does not change in this process.

To distinguish between these two accounts, we considered their effects on response latency, in addition to recall precision. Previous behavioral experiments have shown that response latency increases with set size (Pearson et al., 2014; Schneegans and Bays, 2016), consistent with a process of evidence accumulation to a fixed threshold for response generation (Reddi and Carpenter, 2000; Usher and McClelland, 2001; Gold and Shadlen, 2007). In the decay model, we expect that longer delay durations will likewise lead to increased response latencies, because both set size and delay attenuate the spiking activity for each memorized item. Specifically, if we vary both set size and delay duration in a working memory task, we expect to find a fixed relationship between response latency and precision (Fig. 1*A*). In contrast, the drift model predicts that delay duration should affect recall precision (because greater random drift occurs over longer delays), but not response latencies (because total spiking activity does not change; Fig. 1*B*).

To test these conflicting predictions, we used a spatial working-memory task with saccadic responses, using eye tracking to obtain precise measures of response latency and precision. We extended the neural resource model of Bays (2014) with an accumulation mechanism that generates response latencies, and implemented the two accounts of delay effects within this model. We found that the experimental results were accurately reproduced by a drift model of delay effects, but were inconsistent with the decay account.

## Materials and Methods

### 

#### 

##### Behavioral task.

Ten naive participants (3 males, 7 females; aged 22–37 years, mean 26.8 years) took part in Experiment 1 after giving informed consent in accordance with the Declaration of Helsinki. All participants had normal color vision and normal or corrected-to-normal visual acuity. Stimuli were presented on a 27 inch LCD monitor with a refresh rate of 120 Hz. Participants were seated in front of the monitor with their head stabilized by a forehead and chin rest, maintaining a viewing distance of 60 cm. Eye movements were monitored online at 1000 Hz using an infrared video-based eye tracker (Eyelink 1000 Desktop System, SR Research).

**Figure 2.**
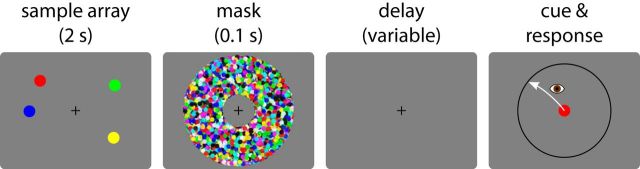
Behavioral task in Experiment 1 (memory condition). Participants fixated a central cross. A circular array of colored disks was presented, followed by a pattern mask. After a variable delay a cue was presented at fixation matching in color one of the items in the sample array, and participants were required to make a saccade to the remembered location of the matching item.

The memory condition of the task is illustrated in Figure 2. Each trial began with the presentation of a central black fixation cross (diameter 0.75° of visual angle). Once stable fixation was achieved (within a radius of 2° around the fixation cross for at least 0.5 s), a memory sample array was presented for a duration of 2 s. The array consisted of one, two, or four saliently colored disks (diameter 1°; color chosen from: red, green, blue, yellow). These were located on an invisible circle with a radius of 6° around fixation, with a minimum angular distance of 30° between disk centers. The presentation of the sample array was immediately followed by a pattern mask display, visible for 0.1 s. This was followed by a delay period (with only the fixation cross visible), whose duration was chosen such that the total memory delay including the mask display was either 0.5, 1, 2, or 4 s.

The fixation cross was then replaced by a centrally presented response cue, in the form of a colored disk that matched one of the disks from the memory sample array (the target). At the same time, a dark gray circle with a radius of 6° around the center appeared, matching the eccentricity of the memory sample stimuli. When this cue appeared, participants had to make a saccadic eye movement to the memorized location of the cued disk from the sample array. Participants were instructed to make this eye movement as precisely as they could, and try to land approximately on the gray circle that served as a guide for saccade amplitude. Once a stable peripheral fixation was achieved following the response saccade (online criterion: fixation held for at least 0.25 s), participants received feedback in the form of a black cross appearing at the saccade endpoint. If central fixation was lost before presentation of the response cue, the trial was aborted and repeated later within the block.

In a control condition, each trial proceeded in the same fashion, except that the memory sample array remained visible throughout the trial. Each participant performed six blocks of 48 trials (288 trials in total) in the memory condition, and two blocks (96 trials in total) in the control condition. Set size and delay duration were varied pseudorandomly and balanced within each block, and memory and control blocks were randomly interleaved.

Ten different naive participants took part in Experiment 2 (3 males, 7 females; aged 18–52 years, mean 27.8 years). This experiment was run on a 21 inch CRT monitor with a refresh rate of 130 Hz, with all other apparatus and setup unchanged. The procedure was the same as in the memory condition of Experiment 1, except for the following modification: the central fixation cross disappeared 0.25 s before the presentation of the response cue (leaving the screen blank during this period). This was intended to give the participants an indication that the response cue was about to appear, to reduce any surprise that might be caused by the early appearance of the response cue at the shortest delays. Each participant completed five blocks of 60 trials in this experiment, with set size and delay duration pseudorandomly varied and balanced within each block.

##### Analysis of eye-movement data.

We smoothed the raw eye-tracking data from each trial with a Butterworth filter, and segmented the data points into a sequence of saccades (minimum peak velocity 50°/s, minimum amplitude 1°) and fixations (minimum duration 0.1 s). We determined the response direction as the angular location of the first stable fixation (minimum duration 0.2 s) after an initial response saccade and potentially subsequent correction saccades following the presentation of the response cue. The response latency was determined as the onset time of the initial response saccade relative to onset of the response cue.

Trials were excluded from analysis if they violated any of the following conditions: the initial response saccade had to occur no earlier than 0.15 s and no later than 2 s after response cue onset, and the amplitude of this saccade had to be no less than 50% and no more than 150% of the sample array eccentricity. If there were correction saccades before a stable fixation was reached, they could not change the direction of the response by >15°. Finally, there could be no blinks before stable fixation was achieved again. We excluded 744 of a total of 3840 trials in Experiment 1 (19.4%) and 645 of 3000 trials (21.5%) in Experiment 2 for failing to meet these conditions.

To focus our analysis on trials in which saccades were directed toward the target item, we fit the three-component mixture model of Bays et al. (2009), which distinguishes between responses directed toward the target, responses directed toward one of the nontarget items, and random responses. Using the method of Schneegans and Bays (2016), we classified as target trials those with a probability exceeding 75% of arising from the target component of the model. Code is available at http://paulbays.com/code/CO16/. Only these trials were included in the analysis of response measures.

##### Experimental design and statistical analysis.

Experiment 1 used a 3 (set sizes: 1, 2, or 4 items) × 4 (delay durations: 0.5, 1, 2, or 4 s) × 2 (task: memory or control) condition within-subjects design, whereas Experiment 2 omitted the different task conditions (resulting in a 3 × 4 within-subjects design). For each subject and each condition, we determined as dependent variables the root mean square deviation (RMSD) of response direction from the target and the median response latency (both based on target responses only). We chose the RMSD instead of e.g., circular SD as a precision measure to improve the robustness of the results (given that only 24 or 25 trials were obtained for each condition in each experiment, and some of these were excluded as lapses or due to their saccade characteristics). We note that all statistical results are unchanged when circular SD or mean absolute error are used as precision measure instead.

We analyzed the effects of set size and delay duration on response precision, response latency, and proportion of target responses using a two-way repeated-measures ANOVA. The behavioral measures of the control condition in Experiment 1 were used as a baseline for any effects of set size and delay unrelated to memory. We subtracted these from the response measures in the memory condition, and applied the same ANOVA to the resulting difference.

##### Neural model.

We aim to provide a unified neural model to explain both response latencies and response distributions in the behavioral task. We use as basis the neural population model proposed by Bays (2014, 2016). In this model, the memorized locations of each of *N* items is represented in a population of *M* spiking neurons with von Mises tuning curves, with total activity normalized across all neurons. The firing rate *r_i_*_,_*_j_* of neuron *i* coding angular location θ*_j_* of item *j* is as follows:

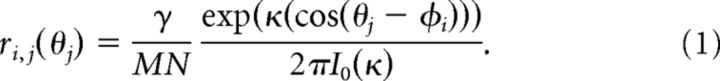
 Here, γ is a gain factor, φ*_i_* is the preferred value and κ the concentration parameter of the neuron's tuning curve, and *I_n_*(·) is the modified Bessel function of the first kind. The number of spikes *n_i_*_,_*_j_* produced by the neuron in a decoding interval *T* is drawn from a Poisson distribution with mean *r_i_*_,_*_j_T*. The report value for the cued item *j* is then decoded from the population spiking activity **n***_j_* by maximum likelihood estimation:

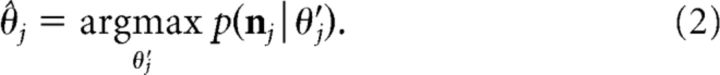
 We extend this model to address saccadic response latencies and associated distributions of saccadic response directions. We assume that for the generation of a saccadic eye movement, spikes from the neural population are integrated to a fixed threshold. The response latency is based on the integration time, and the response direction is based on the spikes produced up to that threshold.

The integration time to a spike threshold *m* is *t* if *m* − 1 spikes occurred in the interval [0, *t*) and one last spike occurs at time *t*. The probability that *m* − 1 spikes occurred within [0, *t*) can be described by a Poisson distribution, based on the constant spike rate $\frac{\gamma }{N}$ in the neural population for a single item. The probability for the final spike to occur at any time *t* is constant with amplitude given by that same spike rate. The distribution of integration times *p*_m_(*t*) can then be described as the product of these two components:

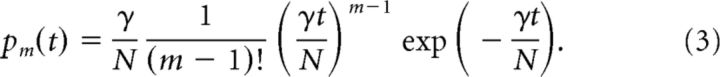
 We assume that the total response latency is composed of the integration time described by the distribution *p*_m_(*t*) plus a random component to reflect factors in saccade initiation that are not directly related to memory retrieval, such as processing of the color cue and execution of the motor plan. We follow Schwarz (2001) in modeling this random component as an exponential distribution *g*(*t*):

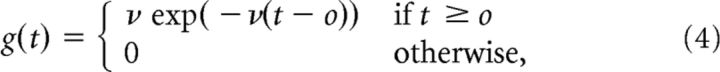
 with rate parameter ν and additional constant offset *o*. The combination of an exponential distribution with a component reflecting the evidence accumulation process has previously been shown to provide close fits to reaction time data in visual search (Palmer et al., 2011). We obtain the final distribution of response latencies, *p_r_*(*t*), by convolving the exponential distribution *g*(*t*) with the distribution of integration times, *p*_m_(*t*):

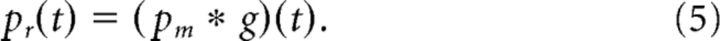
 Examples of the resulting distribution fit to experimental data can be seen in Figure 5*B*.

We assume that saccade direction is based on spikes accumulated until the threshold is reached. However, spikes that are generated early in the integration process are likely to have less influence on the selected movement direction (because their contribution has to be maintained over a longer time until the actual movement is initiated). We model this in a simplified form by assuming that spikes decay with a fixed rate over the integration period (but that this decay does not affect the simpler operation of integration to threshold).

For a single spike occurring at time *t*, the probability that it will have decayed by the end of the integration period, *t*_m_, is described by the cumulative distribution function of the exponential distribution,

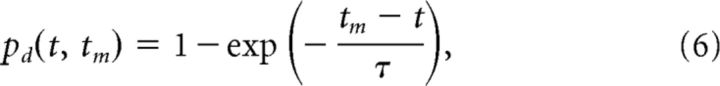
 where τ is the survival rate. For a given integration time *t*_m_, the spike occurrence times for *m* − 1 spikes are uniformly distributed in the interval [0, *t*_m_) (while the last spike must occur at *t*_m_). For each of these spikes, we can obtain the probability that it decays by averaging the above expression for *p_d_* over the possible spike times *t* in the interval [0, *t*_m_). The complement of the resulting expression yields the probability *p_s_* that each spike survives:

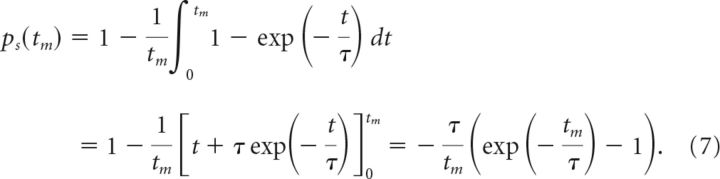
 The number *k* of surviving spikes for a given integration time *t*_m_ then follows a binomial distribution based on the survival probability *p_s_* and the number of spikes *m* − 1. Because the last spike occurring at the end of the integration interval always survives, the resulting number of spikes is one higher than the one obtained from the binomial distribution:

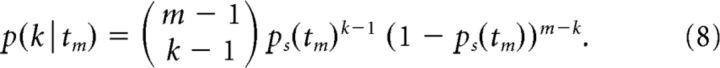
 By integrating over the possible integration times *t*, weighted with the corresponding probability *p*_m_(*t*) from Equation 3, we obtain the distribution of number of spikes *k* that contribute to saccade direction as follows:

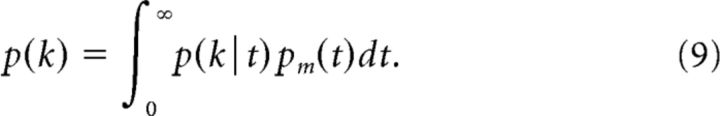
 For each possible number of spikes *k* available for decoding, we can determine a distribution of response errors expected in the neural population model. An analytical formulation for these distributions has been derived by Bays (2016), and we only report the key equations here. Given a neural population that encodes a feature value θ*_j_* as described in Equation 1, the distribution of response errors Δθ̂*_j_* for maximum likelihood decoding from *k* spikes is given by the following:

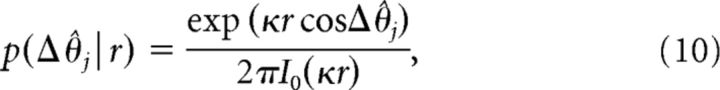
 with

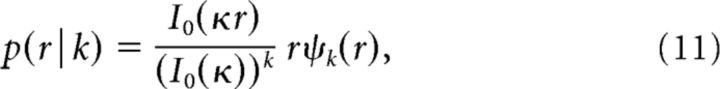
 where *r*ψ*_k_*(*r*) is the probability density function for the resultant length *r* of a uniform random walk of *k* steps (estimated by Monte Carlo methods as by Bays, 2016). We can obtain a distribution of response errors for a given concentration parameter κ and number of spikes *k* by integrating over possible values of *r*. We then obtain the final distribution of response errors as a mixture of these distributions, weighted with the probability *p(k)* from Equation 9:

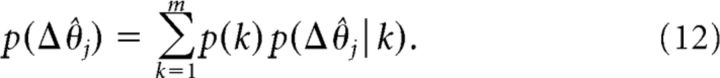
 Examples this distribution fit to experimental data can be seen in Figure 5*A*.

##### Delay effects.

The neural model as described so far addresses effects of set size (through normalization of neural spiking activity across all memorized items), but it does not incorporate any effects of delay duration. We considered two possible mechanisms for delay effects, namely decay of population activity and drift of activity over time.

In the *decay* model, we assume that the gain γ of neural spiking activity is not fixed (as in the original model), but decreases as a function of delay duration *t_d_*. We considered a linear decay with slope ω and lower bound at zero,

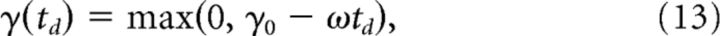
 and an exponential decay with rate λ

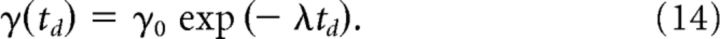
 Distributions of response latencies and response directions are then determined in the model with the gain parameter adjusted for the delay duration in each condition.

In the *drift* model, we assume that the center of mass in population activity changes randomly, following a Brownian motion over the duration of the delay interval, without changing the width or shape of the activity distribution in the population. This Brownian motion of the activity center leads to variability in the currently encoded angular location θ̃*_j_*, which can be described by a von Mises distribution with concentration parameter χ:

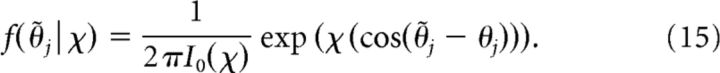
 We considered two model variants again. In the first, the concentration χ is scaled inversely with the delay duration:

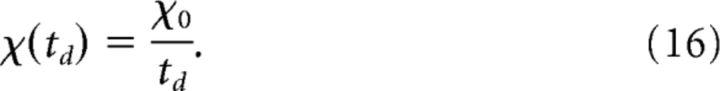
 This reflects the effects of Brownian motion under the assumption of a constant drift rate. In the second variant, we assume that drift rate scales with set size, as has been proposed by Koyluoglu et al. (2017):

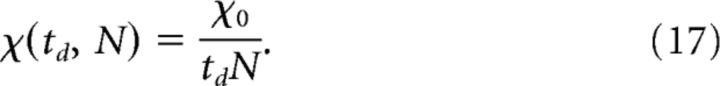
 In both variants, the resulting distribution of response errors is obtained by convolving the response distribution *p*(Δθ̂*_j_*) obtained in Equation 12 with the von Mises distribution *f*(θ̃*_j_*|χ). Importantly, in the drift model, delay duration has no effect on response latencies, since the total number of spikes in the neural population does not change. We note that in all model variants, we only take into account drift or decay during the delay period of the task, and not during the comparatively brief spike accumulation process.

##### Model fitting.

The basic model (without delay effects) has six free parameters, namely gain γ, spike threshold *m*, tuning curve width κ, spike survival rate τ, as well as rate parameter ν and offset *o* for the exponential distribution in the response latency. The two latter parameters only affect response latency measures, whereas κ and τ only affect the distribution of spatial responses. Each variant of the decay/drift mechanism adds one more parameter (ω, λ, or χ_0_, respectively).

We obtained a separate maximum likelihood fit for each individual participant and each model variant. Free model parameters for each participant were shared across all trial conditions, with effects of set size and delay duration captured by inserting the appropriate values for *N* and *t_d_* in the equations above. We first performed a grid search over the parameter space and computed the likelihood of obtaining each participant's actual responses from the model for a large number of parameter combinations. The ranges of parameter values considered here are given in Table 1.

**Table 1.** Parameter values used in grid search and final fit values

Parameter (unit)	Grid range	Grid steps (spacing)	Linear decay	Exp decay	Const drift	Var drift
γ (s^−1^)	(2^3^, 2^9^)	25 (log)	95.2 ± 15.2	98.7 ± 15.9	99.5 ± 19.1	94.2 ± 18.0
*m*	(2^2^, 2^7^)	11 (log)	7.1 ± 0.74	7.0 ± 0.73	7.3 ± 0.95	7.0 ± 0.96
κ	(2^3^, 2^7^)	9 (log)	22.0 ± 2.5	22.2 ± 2.5	26.0 ± 3.5	31.9 ± 5.10
τ (s)	(2^−6^, 2)	8 (log)	0.18 ± 0.093	0.18 ± 0.10	0.14 ± 0.044	0.44 ± 0.17
ν (s^−1^)	(1, 15)	15 (lin)	9.2 ± 0.70	9.3 ± 0.71	9.2 ± 0.70	9.3 ± 0.72
*o* (s)	(0.1, 0.3)	11 (lin)	0.21 ± 0.008	0.21 ± 0.008	0.21 ± 0.008	0.21 ± 0.008
ω (s^−2^)	(2^−2^, 2^4^); 0	13 (log) + 1	0.16 ± 0.13	—	—	—
λ (s^−1^)	(0, *ln*(2))	9 (log)	—	0.017 ± 0.013	—	—
χ_0_ (s^−1^)	(2^7^, 2^14^); ∞	15 (log) + 1			3415 ± 1082	4637 ± 1484

The parameters are gain of spiking activity γ, spike threshold *m*, neural tuning curve width κ, spike survival rate τ, rate parameter ν, and offset *o* for the exponential component of the latency distribution, decay rate ω (linear decay model) or λ [exponential (Exp) decay model], and drift rate χ_0_ (drift models). For each parameter we report the range over which it was varied in the grid search (with an additional discrete value reflecting no delay effects for ω and χ_0_), the number of steps in the grid search and their spacing within the given range (logarithmic or linear), and the maximum likelihood estimates (mean over participants ± SE) for each of the four model variants. Threshold values *m* are always rounded to the nearest integer. The fit values for χ_0_ exclude one outlier in the constant (Const) drift variant (with χ_0_ = 8.3 × 10^7^ s^−1^), and one outlier in the variable (Var) drift variant (with χ_0_ = 2.4 × 10^6^ s^−1^), reflecting individual model fits with virtually no drift.

To make the grid search feasible for the large number of parameters, we made use of the fact that response latencies and response directions are each affected only by a subset of all parameters, so the likelihoods can be computed independently and then combined. We then refined the fits by running an optimization algorithm (Nelder–Mead method, function *fminsearch* in MATLAB), using the optimal parameters from the grid search as starting point.

## Results

### Experiment 1

In the memory condition of Experiment 1, participants viewed a sample array of colored disks, with set size varying between one and four items. After a variable delay, participants were cued with a color and performed a saccadic eye movement to the memorized location of the matching disk. The angular location of the first stable fixation after the response saccade and the response latency were determined as primary response measures for each trial.

We fit participants' response distributions with a three-component mixture model (Bays et al., 2009), yielding an estimate of 97.4% of responses directed to the target item, 2.2% swap errors (directed to one of the nontarget items), and 0.4% random responses (averaged over all set sizes and delay durations). We then used these mixture model fits to classify individual trials into those with responses to the target and those with lapse (nontarget or random) responses. Because stimuli in the sample array were well separated and saccadic responses were relatively precise, almost all trials could be classified unambiguously (for >99% of all trials, the estimated probability of being a target trial was either >0.95 or <0.05).

The classification result showed that for set sizes one and two, almost all responses were directed to the target (99.8 and 99.3%, respectively), whereas the proportion was slightly lower at set size 4 (93.1%). This effect of set size was significant (two-way repeated-measures ANOVA, set size: *F*_(2,18)_ = 5.6, *p* = 0.013), whereas the effects of delay and the interaction between them did not reach significance (delay: *F*_(3,27)_ = 2.5, *p* = 0.083; interaction: *F*_(6,54)_ = 2.1, *p* = 0.065). It is possible that the distributions of response errors and response latencies differ between target and lapse trials, so for further analysis, we include only target trials to allow a fair comparison of these metrics between conditions.

**Figure 3.**
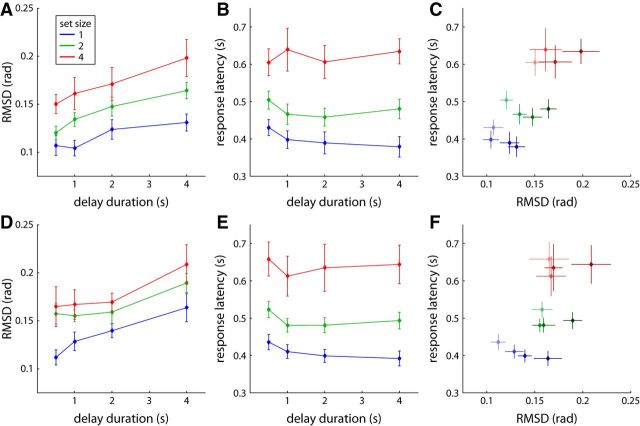
Behavioral results for Experiment 1 (memory condition) and Experiment 2. Response measures are shown for trials classified as responses to target only. ***A***, RMSD of saccadic response direction from target direction for Experiment 1. ***B***, Median saccadic response latency for Experiment 1. ***C***, Median response latency plotted against RMSD for each set size and delay condition. Data points for different delay durations are plotted in different shades, with brighter colors indicating shorter delays. ***D***–***F***, Corresponding results for Experiment 2. Error bars indicate ±1 SE in all panels.

Figure 3, *A* and *B*, shows the results for response error (measured as RMSD) and median response latency of target trials for all set sizes and delays. Response error can be seen to increase with both set size and delay duration. Both of these effects were statistically significant, with no significant interaction (set size: *F*_(2,18)_ = 23.4, *p* < 0.001; delay: *F*_(3,27)_ = 9.6, *p* < 0.001; interaction: *F*_(6,54)_ = 0.34, *p* = 0.91). Median response latency visibly increases with set size, but critically, increasing the delay duration did not cause longer response latencies. Statistical analysis confirms a significant effect of set size (*F*_(2,18)_ = 46.5, *p* < 0.001), but not of delay duration (*F*_(3,27)_ = 2.2, *p* = 0.11), and no significant interaction (*F*_(6,54)_ = 1.7, *p* = 0.15).

One surprising aspect in the response latency results is that median response latency is actually higher (i.e., responses are slower) for the shortest delay duration compared with longer delays, at least at set sizes 1 and 2. This effect is not predicted by either model of delay effects considered here, and is unlikely to arise from the working memory recall process. We hypothesize that it is instead an effect of surprise or lack of preparedness if the cue is presented very quickly after the offset of the memory array (Niemi and Näätänen, 1981), given that delay conditions were mixed within blocks and not predictable for the participant. It is possible that this effect masks an actual increase of the time required to retrieve the target location from memory for longer delays. To rule this out, we compared the results from the memory condition with the results from a control condition in which the sample array remained visible throughout the trial.

We analyzed the results of the control condition in the same way as the memory condition, including only target trials (99.0% of all trials). We found a similar effect of higher response latencies at the shortest delay, confirming the assumption that this is not a memory effect. We subtracted the median response latencies in the control condition from those in the memory condition, separately for each combination of set size and delay duration. We reasoned that this would eliminate all effects of preparedness on the result, because these should be the same in memory and control conditions. We still found a significant effect of set size, and not of delay duration or interaction of both factors, on median response latency differences (set size: *F*_(2,18)_ = 11.2, *p* < 0.001; delay: *F*_(3,27)_ = 1.2, *p* = 0.35; interaction: *F*_(6,54)_ = 0.90, *p* = 0.50). Response errors in the control condition did not show systematic variation with either set size or delay, and subtracting these from the results of the memory condition did not alter the significant effects of set size and delay found here (set size: *F*_(2,18)_ = 30.2, *p* < 0.001; delay: *F*_(3,27)_ = 10.7, *p* < 0.001; interaction: *F*_(6,54)_ = 0.58, *p* = 0.75).

The combination of an increasing SD with no systematic change in response latency for longer delay durations is consistent with the drift model, but not the activity decay model, of delay effects. This is further illustrated in Figure 3*C*, where we plot response latency against response RMSD for each set size and delay duration. The plot matches the qualitative prediction of the drift model (Fig. 1*B*). In particular, it shows that response latency can vary greatly for different conditions that have almost the same response error. This directly contradicts the prediction of the activity decay model that there should be a fixed relationship between these two variables across all conditions.

### Experiment 2

In Experiment 2, we sought to address the issue of different levels of preparedness at different delay durations through a modification of the experimental design. The fixation point disappeared briefly before the presentation of the cue, giving participants an indication that they would be required to make a response. We applied the same analyses as for the memory condition in Experiment 1. The mixture model yielded an estimate of 97.6% target responses, 1.8% swap errors, and 0.6% random guesses (averaged over all conditions). In the classification of individual trials, the proportion of target trials was 99.8% at set size 1, 99.5% at set size 2, and 95.5% at set size 4 (98.3% over all valid trials). The effect of set size on proportion of target trials was significant (*F*_(2,18)_ = 4.7, *p* = 0.022), whereas there was no significant effect of delay (*F*_(3,27)_ = 1.6, *p* = 0.21) and no significant interaction (*F*_(6,54)_ = 1.3, *p* = 0.28).

Analyzing only the target trials, we found the same overall pattern of response errors as in Experiment 1, shown in Figure 3*D*. There were significant effects of set size (*F*_(2,18)_ = 12.3, *p* < 0.001) and delay (*F*_(3,27)_ = 13.0, *p* < 0.001), and no interaction (*F*_(6,54)_ = 0.24, *p* = 0.96). The modification of the task procedure did not eliminate the higher response latencies for the shortest delay duration of 0.5 s, which were still apparent at all set sizes (Fig. 3*E*). There was a significant effect of set size on median response latencies (*F*_(2,18)_ = 24.2, *p* < 0.001), while the effect of delay failed to reach significance (*F*_(3,27)_ = 2.8, *p* = 0.060), with no significant interaction (*F*_(6,54)_ = 0.63, *p* = 0.70). The tendency for an effect of delay duration is driven only by the higher response latency at the shortest delay, and disappears if we exclude this condition from the analysis (*F*_(2,18)_ = 0.18, *p* = 0.84). Plotting response latencies against response errors (Fig. 3*F*) produces the same general pattern as in Experiment 1, matching the prediction of the drift model of delay effects and inconsistent with the activity decay model.

### Model fits

We used a neural model to fit response error and response latency data, and to compare possible mechanisms underlying delay effects in working memory. We used the same model to fit data from both Experiment 1 (memory condition only) and Experiment 2, given that the small modification in experimental procedure did not qualitatively change behavioral measures.

The neural resource model assumes that memorized stimulus locations are encoded in populations of spiking neurons, and responses are generated by maximum likelihood decoding from the spiking activity (Bays, 2014, 2016; Schneegans and Bays, 2017a). We assume that for the generation of a saccadic eye movement, total spiking activity in the population is integrated to a fixed threshold. Response latencies are based on the integration time, and the response direction is based on the generated spikes up to that threshold. Poisson noise in neural spiking activity leads to deviations between response value and encoded value and to variability in response times. Critically, total spiking activity in the model is normalized across all memorized items, a mechanism that has successfully accounted for set size effects in visual working memory in previous work (Bays, 2014).

Within this basic model, we implemented two different hypotheses for the mechanism underlying effects of delay duration in working memory. In the decay model, spiking activity in the neural populations continuously decreases over time, following either a linear or an exponential decay. In the drift model, total activity levels remain fixed, but the active regions within the populations undergo random drift over time, following a Brownian motion. We considered either a fixed rate of drift across all conditions, or a drift rate that is scaled with the number of items (more drift at higher set sizes). We obtained a maximum likelihood fit for each model variant for the combined response latency and response direction results for each individual participant (pooled over Experiments 1 and 2), and compared the models based on the summed log likelihood of the experimental data under the fitted model (given that all variants had the same number of free parameters).

**Figure 4.**
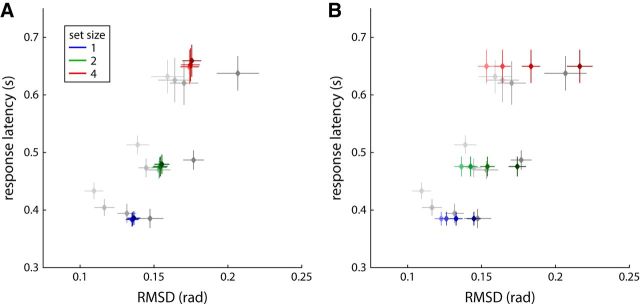
Model fits and experimental results, pooled over Experiments 1 and 2. All results are plotted as median response latency against RMSD for each set size and delay duration (as in Fig. 3*C*,*F*). Behavioral results are plotted as gray diamonds, model results as colored diamonds, with lighter shading of data points indicating shorter delay duration. ***A***, Model with exponential decay of activity over delay period. ***B***, Model with set-size-dependent drift over delay. Error bars indicate ±1 SE in all panels.

For the decay model, the variant with linear decay attained a total log likelihood of 5238, whereas the variant with exponential decay attained a value of 5244 (Δlog(*L*) = 6.6 in favor of the exponential decay variant). The parameters of the best fits for both models are reported in Table 1. Figure 4*A* (colored symbols) shows the median response latencies plotted against the response errors of the model fits for the variant with exponential decay, together with the corresponding experimental results for comparison (gray symbols; pooled over participants from both experiments). The model captures the effects of set size reasonably well, but entirely fails to reproduce the delay effects observed in the experimental data.

The set size effects are accounted for in the neural model through the normalization of total spiking activity. At higher set sizes, fewer spikes are generated in the population representation for each individual item, leading to longer integration times. We further assumed in the model that spikes occurring earlier in the integration period contribute less reliably to the response, so that fewer spikes are effectively available for the decoding of the memorized object location at higher set sizes.

Previous implementations of the neural population model assumed a fixed time window for decoding (Bays, 2014), which creates the same qualitative effect of an inverse relationship between set size and number of spikes, but cannot produce variations in response latencies. We introduced the integration to a fixed spike threshold to fit latency data, but in the original formulation (without discounting of earlier spikes), this would have undesired effects: the model would produce the same decoding precision independent of the spike rate for each item (because the total number of spikes for decoding would always equal the threshold), and the model would be capable of producing arbitrarily high precision by using a higher threshold. The latter aspect in particular is unrealistic given that the decoding process in the neural system is itself affected by random noise. The discounting of earlier spikes in decoding the memorized feature was introduced as a simple mechanism to reflect this noise. We note that this implementation with a fixed threshold is not necessarily appropriate to model typical cued recall tasks, in which subjects adjust a probe stimulus without time constraints to make their response.

The decay model assumes that the effect of delay duration is caused by the same basic mechanism that generates the set size effect, namely a decrease in spiking activity for each item. The data points generated by the model for different combinations of set size and delay duration must therefore lie on a single line, because they vary only in a single parameter. The experimental data does not match this pattern, and the fit must therefore remain poor. Indeed, for the majority of participants, the best fit showed almost no decay of activity at all (decrease of <1% of maximum gain over the maximum delay in 16 of 20 participants for the linear decay variant, and for 18 of 20 participants for exponential decay).

For the drift model, the variant with a set-size-dependent drift showed overall higher log likelihood values than the variant with fixed drift rate (5295 vs 5275, difference Δlog(*L*) = 20.1). The parameters of the best model fits are given in Table 1. Importantly, these fits show robustly higher likelihood values than either of the decay models (Δlog(*L*) = 51.2 for the better fitting variant of each model, with higher fit likelihood for 17 of 20 participants).

The fits for the model with set-size-dependent drift are shown in Figure 4*B*, and can be seen to capture the key characteristics of the behavioral results. The drift of neural activity does not affect overall spike rate in the neural population, such that response latency in this model does not vary with delay duration. This matches the behavioral results, where we did not find a significant effect of delay on response latency in either experiment. The effect of set size on response latency is a result of the normalization of total activity across memory items, as described for the decay model. The set-size-dependent drift based on Brownian motion qualitatively captures the effects of delay on response errors. It should be noted that this drift mechanism also contributes to the effects of set size on response errors, making the mechanism of discounting early spikes in the basic model partly redundant.

**Figure 5.**
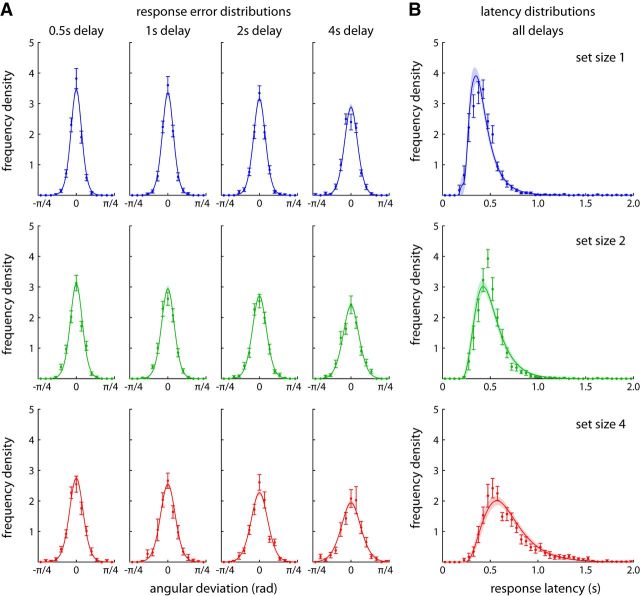
Distributions of response errors and latencies for experimental data and model fits. ***A***, Distributions of deviations of the response direction from the target direction, shown separately for each set size and delay duration. ***B***, Distributions of response latencies, plotted separately for each set size, pooled over delay durations. Experimental data are shown as points with error bars indicating ±1 SE; mean fits of the set-size-dependent drift model are shown as solid lines with shaded region indicating ±1 SE.

Figure 5 shows the distributions of response errors and latencies in the experimental data, and fits from the model with set-size-dependent drift. Distributions of angular response deviations from the target are shown in Figure 5*A* separately for each set size and delay duration. Distributions can be seen to become broader with both set size and delay duration, and these effects are quantitatively captured by the model fit.

Response latency distributions are shown in Figure 5*B* for different set sizes, collapsed across delay conditions (because response latencies did not differ significantly with delay in the experimental data, and delay duration does not affect response latency distributions in the drift model). The model qualitatively captures the shape of the latency distributions and the effects of set size, although some deviations between fits and experimental results are noticeable. In particular, the model fit for set size 2 appears broader than the experimentally observed latency distribution.

This discrepancy is likely due to a certain unpredicted property of the experimental results: whereas median response latency is significantly higher at set size 2 than set size 1 (paired *t* test for data pooled over delay conditions and both experiments; *t*_(19)_ = 9.7, *p* < 0.001), the SD of response latencies within participants did not differ significantly between these conditions (*t*_(19)_ = 1.0, *p* = 0.32), and was numerically slightly lower at set size 2. This pattern also holds when comparing each delay condition separately (median latency significantly greater for set size 2 than set size 1, all *p* < 0.001, latency SD not significantly different; all *p* > 0.13).

It is not possible to account for this pattern with integrator or drift-diffusion models of decision making, where an increase in median latency is necessarily accompanied by an increase in latency variability under otherwise equal conditions. This aspect of the data can therefore not be captured by the model, in which the latency distributions for different set sizes are determined by shared parameters. We conjecture that the experimental observation may be due to the task being conceptually somewhat different at set size 1, in that participants do not have to use the color cue to select an item from working memory in this condition.

## Discussion

We have considered two possible ways in which delay duration could affect neural population activity in working memory tasks, and derived specific behavioral predictions from them. If a decay of activity occurs over delay, this should produce both an increase in response error and response latency for memory recall, with a fixed relationship between these two measures when both set size and delay duration are varied in a cued recall task. Conversely, if delay produces drift in population codes without decrease in activity levels, response latencies should remain unchanged, whereas response errors increase with longer delays. Behavioral results from a spatial working memory task with saccadic response qualitatively matched the predictions of the drift model. Quantitative fits with a neural model implementing both forms of delay effects confirmed that the drift model can account significantly better for the behavioral data, and that it can successfully reproduce the response error and latency distributions observed in the task.

We focused our analysis on those trials in which responses were directed to the cued target item, using a recently developed statistical method to exclude lapses, i.e., swap errors and random responses (Schneegans and Bays, 2016). The target trials are most informative to distinguish between the two models of delay effects, and the exclusion of lapse trials avoids confounding effects arising from different latency and precision distributions in target and lapse responses. One previous study suggested that delay duration affects recall performance specifically through “sudden death” of memory representations for individual items, without affecting recall precision for other items (Zhang and Luck, 2009). Here, we found clear evidence for a decrease in recall precision for remembered items, consistent with findings of Pertzov et al. (2017) and at least partially supported by the results of Shin et al. (2017). Like Pertzov et al. (2017), we also observed that a large proportion of lapse responses could be explained by swap errors, a possibility not considered by Zhang and Luck (2009). This calls into question the interpretation that they are evidence for “sudden death” of individual memory representations.

We note that the population model used here is not designed to produce swap errors, because it does not capture the bindings of colors to locations that is required to select the target location given a color cue. A previous extension of the model with conjunctive coding has demonstrated that the neural population coding can account for swap errors as well (Schneegans and Bays, 2017a). Applying this to the current task would mean extending the spike integration mechanism also, to allow the selection of different response options (Brown and Heathcote, 2008). This is beyond the scope of the current work.

A key finding of the present study was that response latencies for responses to the target do not increase with delay duration. In contrast, changes in set size produced a robust effect on response latencies in both experiments, demonstrating that the used method is sensitive enough to detect systematic changes in latency. In particular, we observed that different combinations of set size and delay duration that produced very similar response errors nonetheless showed strong differences in response latencies (which depended on set size only). This directly contradicts the decay model's prediction of a fixed relationship between response errors and latencies. The finding is consistent, however, with the drift model, which assumes that the precise value encoded in the population activity changes over time, whereas the amplitude and shape of the population activity remain the same (and therefore response latencies are unaffected).

Some earlier working memory studies had reported an increase of response latencies with delay duration (Phillips and Baddeley, 1971; Paivio and Bleasdale, 1974). However, these studies used change detection paradigms, and therefore could not distinguish between the times for memory retrieval and decision making. Likewise, several studies failed to detect any decrease of working memory performance with longer delay durations, but these used either discrete or highly discriminable stimuli with categorical responses (Morris, 1987; Kahana and Sekuler, 2002; Lilienthal et al., 2014), and therefore were insensitive to small changes in memorized features. In contrast, the current experimental paradigm with free saccadic responses allows both a direct estimation of memory retrieval time and a detection of graded changes in memory content.

In the present work, we did not aim to capture the neural processes that may generate drift or decay over the delay duration, but rather provide a parametric description of the considered delay effects. This enables us to test the plausibility of different changes in population activity independent of the specific underlying mechanism. The drift mechanism supported by our results is, however, traditionally associated with attractor models of working memory (Wang, 2001; Chaudhuri and Fiete, 2016). In these models, activity that is induced in a neural population by external stimulation is sustained through excitatory recurrent connections, balanced by longer-range inhibition. Random drift of self-sustained activity bumps over time is a general feature of this class of models, which has been described in various implementations using either rate coding (Camperi and Wang, 1998; Schneegans et al., 2014) or spiking neurons (Compte et al., 2000; Wei et al., 2012).

The drift in these models shows the same general features as assumed in our parametric implementation: it is caused by small variations in activity due to random noise that are propagated over time (because the future activity state depends only on the current one), leading to a form of random walk of the activity bump's position; and it does not change the shape or amplitude of the activity bump, because these are determined only by the balance of excitation and inhibition (Amari, 1977; Compte et al., 2000). Importantly, evidence for drift of neural activity over time consistent with these models has also been found in an electrophysiological study in macaque monkeys, using a spatial working-memory task similar to the one-item condition in the present work (Wimmer et al., 2014).

The best fitting model for the present experimental results assumed that drift rate scales with set size (although we note that we did not find a significant interaction between set size and delay duration in the analysis of response errors in the behavioral data). In attractor models, the interaction between drift and number of encoded items depends strongly on implementation details. The model of Koyluoglu et al. (2017) showed drift rates increasing with set sizes in two different implementations (either assuming that neural resources are divided evenly across items or using a recoding of memoranda before the retention stage), and produced close fits for the behavioral data from Pertzov et al. (2017). In the model of Schneegans and Bays (2017b), using spatially unspecific inhibition between items, no change in the amount of random drift was observed between set sizes 1 and 2. Almeida et al. (2015) used a spiking neuron model with broad, but localized inhibition, and they likewise observed no change in random drift with set size. However, their model predicted that interactions between memorized items would generate directed drift in the form of attraction or repulsion (dependent on feature similarity; Johnson et al., 2009), and consequently an overall increase in response variability with set size. The authors confirmed these predictions in behavioral experiments.

As an alternative to sustained activity models, it has been proposed that stimuli in working memory tasks may be encoded in an activity-silent state, e.g., through rapid synaptic plasticity (Lewis-Peacock et al., 2012; Barak and Tsodyks, 2014; Stokes, 2015; Rose et al., 2016), and be reactivated by unspecific input at recall. In such a memory state without sustained activity, there can be no drift of encoded positions over time as described in attractor models. The decrease in recall performance observed in the current study would have to be accounted for by fading of the learned synaptic connections, which we expect to produce effects largely identical to the decay account described here. Such models can therefore not explain the decrease in recall precision without change in response latencies observed here.

Another account of delay effects in working memory is offered by interference models (Lewandowsky et al., 2009; Oberauer et al., 2012). Souza and Oberauer (2015) argued that longer delays affect performance only by decreasing temporal distinctiveness between the current sample array and the array memorized for the preceding trial. In so far as such an account assumes that low temporal distinctiveness leads to intrusion of preceding items in response generation, it can be refuted by the current approach: such intrusions should either (1) be observed as erroneous reports of previous items, which would have been excluded as lapses in the present study, so cannot contribute to the changes we observed in memory precision; or (2) result in changes equivalent to an increase in set size: a prediction that was explicitly refuted in the present results. It is possible, however, that interference from previous trials has detrimental effects without specific intrusions of earlier items (Dillon and Thomas, 1975). Testing this possibility will require more specialized experimental designs and is beyond the scope of the current study.
